# A Heterogeneous Sensing System-Based Method for Unmanned Aerial Vehicle Indoor Positioning †

**DOI:** 10.3390/s17081842

**Published:** 2017-08-10

**Authors:** Can Wang, Kang Li, Guoyuan Liang, Haoyao Chen, Sheng Huang, Xinyu Wu

**Affiliations:** 1 Guangdong Provincial Key Laboratory of Robotics and Intelligent System, Shenzhen Institutes of Advanced Technology, Chinese Academy of Sciences, Shenzhen 518055, China; can.wang@siat.ac.cn (C.W.); gy.liang@siat.ac.cn (G.L.); sheng.huang.siat@gmail.com (S.H.); 2 School of Optical and Electronic Information, Huazhong University of Science and Technology, Wuhan 430000, China; likang@hust.edu.cn; 3 School of Mechanical Engineering and Automation, Harbin Institute of Technology Shenzhen Graduate School, Shenzhen 518055, China; hychen5@hitsz.edu.cn; 4 Department of Mechanical and Automation Engineering, The Chinese University of Hong Kong, Hong Kong 999077, China

**Keywords:** unmanned aerial vehicle, indoor positioning, heterogeneous sensing system, data fusion

## Abstract

The indoor environment has brought new challenges for micro Unmanned Aerial Vehicles (UAVs) in terms of their being able to execute tasks with high positioning accuracy. Conventional positioning methods based on GPS are unreliable, although certain circumstances of limited space make it possible to apply new technologies. In this paper, we propose a novel indoor self-positioning system of UAV based on a heterogeneous sensing system, which integrates data from a structured light scanner, ultra-wideband (UWB), and an inertial navigation system (INS). We made the structured light scanner, which is composed of a low-cost structured light and camera, ourselves to improve the positioning accuracy at a specified area. We applied adaptive Kalman filtering to fuse the data from the INS and UWB while the vehicle was moving, as well as Gauss filtering to fuse the data from the UWB and the structured light scanner in a hovering state. The results of our simulations and experiments demonstrate that the proposed strategy significantly improves positioning accuracy in motion and also in the hovering state, as compared to using a single sensor.

## 1. Introduction

A GPS-based positioning system is a nearly perfect approach to UAV positioning in an outdoor environment, and it is widely used for many kinds of devices, including smartphones, self-driving cars, and so on. While the application is restricted in an indoor environment, it is hard to find a satisfying alternative sensor to provide accurate positioning information inside. Because of this, research into indoor localization systems is becoming more and more popular, and plays an important role in many complex applications of unmanned ground and aerial vehicles [1]. We can apply an indoor localization system to find the location of specified products in warehouses [2] or of firemen in a building, to assist in the automatic driving system of an unmanned vehicle, and to help blind people walk inside a building [3,4].

**Figure 1:**
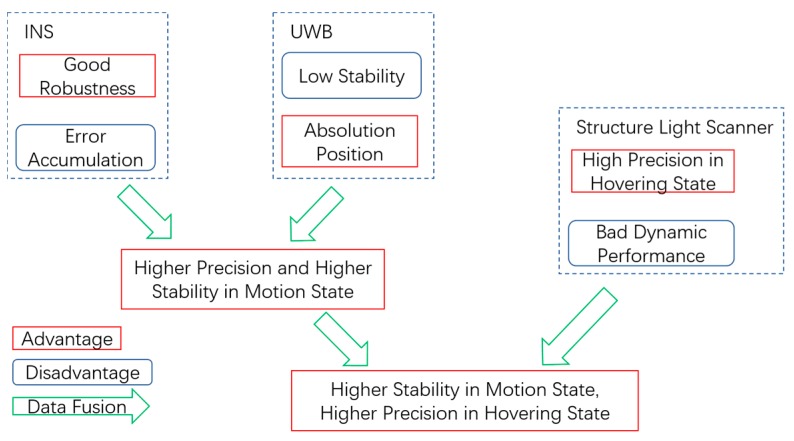
The advantages and disadvantages of a heterogeneous sensing system.

In some realistic applications, for example, to carry a box with a UAV requires that the positioning accuracy of the UAV must be on the order of centimeters. However, designing and implementing such an indoor positioning system for a UAV with that level of accuracy would be difficult and expensive. On the other hand, the decimeter level of positioning accuracy is sufficient for vehicles in motion. In this article, we proposed a low-cost heterogeneous sensing system based on INS, UWB, and a structured light scanner to solve the above problem. While the UAV is in motion, positioning accuracy at the level of centimeters is implemented through the integration navigation of the INS and UWB. When the UAV is executing tasks, a structured light scanner is introduced to provide more accurate positioning. In this case, the structured light scanner works only when the vehicle is in a hovering state. Figure 1 summarizes the advantages and disadvantages of the heterogeneous sensing system. With the fusion of the INS, UWB, and structured light scanner, it is possible to realize higher stability as well as precision for an apparatus in both the moving and hovering states. Although there are other technologies that can provide higher accuracy, the proposed method stands out for its all-round performance in both the motion and hovering state and is a low-cost solution in respect to the application of UAV.

We applied adaptive Kalman filtering to fuse the data from the INS and UWB while the UAV is in motion. The accumulated errors of the INS will be corrected by the absolute positioning data from the UWB. While the UAV is hovering in the specified area, the structured light scanner will start to work as an auxiliary device to retrieve more accurate positioning information. The data fusion strategy integrates the positioning data measured by three different sensors into a consistent, accurate, and useful representation in this paper.

The main contributions of this paper include the following: (1) A self-made structured light scanner based on structured light, which is used to improve the positioning accuracy of an apparatus in a hovering state; (2) Adaptive Kalman filtering to fuse the data of the INS and UWB while in motion; (3) Gauss filtering to fuse the data of the UWB and the structured light scanner in a hovering state. Moreover, we conducted experiments and simulations to verify the effectiveness of the proposed strategy.

In the following section, we introduce the setup of the hardware platform and analyze the mathematical model of different components in detail. Then, the optimized data fusion strategy is described, including the fusion of the INS and UWB in motion and the fusion of the UWB and structured light scanner in a hovering state. Finally, the performance of the proposed strategy is verified by simulations and experiments, and the results show that with the complement of the structured light scanner, the UWB and the INS, a micro UAV is able to achieve indoor positioning with high precision over a long distance.

## 2. Related Work on Heterogeneous Sensing Systems

Indoor localization technology provides robots with the ability to finish tasks automatically by detecting the location of an object. High-precision and robust indoor navigation remains a difficult task, although new technologies have developed rapidly [5,6] in recent decades. The development of indoor localization system benefits from the rapid growth of related sensors. Various sensors are utilized for indoor positioning tasks, such as camera work, laser Lidar, wireless sensing networks, IMU (Inertial Measurement Unit), and optical flow [7,8,9]. Girish et al. integrated the data from an IMU and a monocular camera to accomplish indoor and outdoor navigation and control of unmanned aircraft [10]. Wang et al. designed a comprehensive UAV indoor navigation system based on INS, optical flow, and scanning laser Lidar, and the UAV, thus enhanced, was able to estimate the position accurately [11]. Huh et al. described an integrated navigation sensor module including a camera, a laser scanner, and an inertial sensor [12]. Tiemann et al. build a UWB indoor positioning system, which enabled the autonomous flying of the UAV [13]. Generally speaking, methods based on a camera, which are usually sensitive to changes in lighting conditions and environment, are rarely robust. Methods based on wireless positioning technology are unstable in complex environments. Therefore, positioning by means of a single sensor rarely yields a satisfying result. In this paper, we focus on a setup based on a combination of INS, UWB, and a structured light scanner that is expected to provide improved positioning capability.

INS is a navigation system that uses the IMU to track the velocity, position and orientation of a device. Development of Micro-Electro Mechanical System (MEMS) technology has made it possible to easily place a small and low-cost IMU on the UAV platform. INS can provide high precision navigation for a short period of time. As the time increases, the accuracy declines rapidly because of the drift error accumulations caused by inherent defects [14].

A UWB wireless sensor network is a promising technology for indoor real-time localization. An indoor wireless positioning system consists of at least two separate hardware components: a signal transmitter and a measuring unit. The latter usually carries out the major “intelligence” work [15]. The measuring unit of the UWB makes use of Time of Flight (TOF), and its large bandwidths enable high precision measurement [16]. An obvious difference between a conventional wireless network and a UWB is that the former transmits information by varying the power level, frequency, or phase; the latter transmits information by generating radio energy at specific time intervals and occupying a large bandwidth, thus enabling the positioning service. A UWB technique could provide a localization service with high precision and low power consumption. Note, however, that in some complex indoor environments, a UWB system will be influenced by various multipath phenomena, especially in non-line-of-sight conditions.

Many indoor navigation robots use line structured light vision measurement systems for mapping, localization, and obstacle avoidance [17,18]. We have designed a structured light scanner based on triangulation technology with a camera, a low-cost light laser and some electronic devices. An important step in achieving this was to configure the light’s medial axis (also called the skeleton) with subpixel accuracy. Skeletonization provides an effective and compact representation of an object by reducing its dimensionality to a “media axis” or “skeleton,” while preserving the topology and geometric properties of the object [19]. An object in two dimensions is transformed into a curved skeleton containing one-dimensional structures.

A low-cost light stripe laser is not expected to provide the superior performance, (e.g., little laser beam width, divergence angles and beam propagation ratios) of which a high-quality industrial laser is capable, especially when the laser projects a light stripe on a rough surface or a surface with a high reflection rate. The camera will capture a fuzzy light stripe with strong diffuse reflection, or specular reflection inevitably will occur. Thus, a theory called the fuzzy distance theory (FDT), which is designed to deal with fuzzy objects, is adapted here to solve the problems caused by fuzzy light stripes.

Distance Transform (DT) is a process that iteratively assigns a value at each location within an object that is the shortest distance between that location and the complement of the object. However, the notion of a hard DT cannot be applied to fuzzy objects in a meaningful way. The notion of DT for fuzzy objects, called fuzzy distance transform (FDT), becomes more important in many imaging applications, because we often deal with situations that involve data inaccuracies, graded object compositions, or limited image resolution [20]. In general, FDT is useful, among other things, in feature extraction, determination of local thickness or scale computation, skeletonization, and the analysis of morphological and shape-based objects. Over the past few decades, DT has been popularly applied to hard objects only. Most DT methods approximate global Euclidean distance by propagating local distances between adjacent pixels.

INS, UWB, and a structured light scanner provide position information independently, but each has advantages and disadvantages. INS is used to quickly track the position of an object relative to a known starting point with high precision, but it suffers from accumulations of error. UWB could provide absolute positioning information, but its results are unstable due to the complexities of the indoor environment. A structured light scanner is capable of achieving centimeter-level accuracy, but it only works while the vehicle is hovering in a specified area. In order to utilize the above information effectively, a heterogeneous sensing system is constructed, and an optimized data fusion strategy is designed to fuse information gleaned from different sensors. Data fusion is a technology that combines information from several sources in order to form a unified result, which has several advantages, including enhancing data authenticity or availability. Data fusion technology is now widely deployed in many fields such as sensor networks, unmanned ground and aerial vehicles, robotics and image processing, and intelligent systems [21,22,23]. Generally, the data fusion of multi-sensor devices provides significant advantages over single-source data, and the combination of multiple types of sensors may increase the accuracy of result.

## 3. Hardware Setup

**Figure 2:**
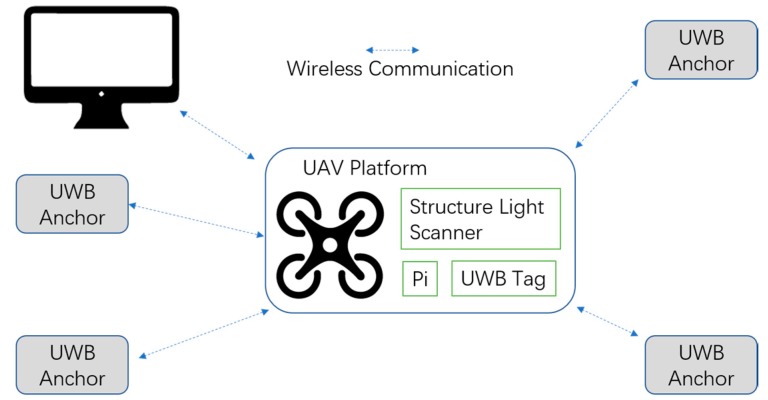
Hardware setup.

**Figure 3:**
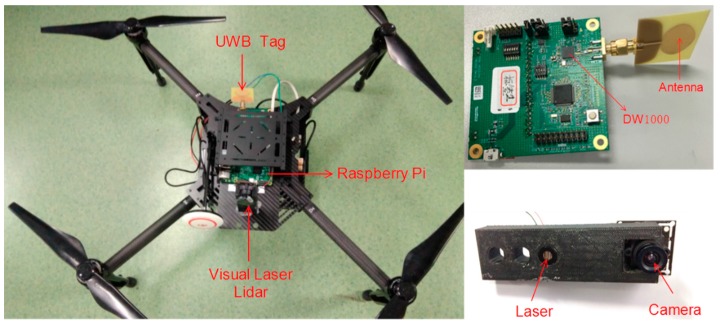
UAV (**left**), UWB (**upper right**) and structured light scanner (**lower right**).

The hardware platform that we used is composed of a PC (with OS Windows 10, CPU i5-7300HQ, Memory 8G DDR3), a micro UAV (DJI M100, DJI-Innovations, Shenzhen, China), a Raspberry Pi 3B, IMU (integrated in the UAV), a UWB (DW1000), and a structured light scanner (self-made) that can obtain position information along the x and y axes. The system architecture is illustrated in Figure 2. The main components are presented in Figure 3. The computation work was finished using a Raspberry Pi, except that the algorithm with the structured light scanner was calculated on the PC and transmitted to the Raspberry Pi through a Wi-Fi connection.

A nine axis IMU is integrated in the micro UAV, including three mono-axial accelerometers that allow for the detection of the current rate of acceleration; three mono-axial gyroscopes for the detection of changes in rotational attributes like pitch, roll, and yaw; and three magnetometers for the detection of the orientation of the body.

The UWB module is configured with DW1000, a fully integrated low power single chip CMOS radio transceiver IC compliant with the IEEE 802.15.4-2011 ultra-wideband (UWB) standard. The module supports a two-way ranging exchange. There are four anchor sensors, the positions of which are known, and they are used for station and other tag sensors placed on the UAV that are used for positioning. The system allows for the location of objects in real time location systems (RTLS) to a precision of 15 cm indoors and achieves an excellent communications range of up to 150 m, thanks to coherent receiver techniques. The application of a UWB in complex environments is still a challenge. Due to the effect of NLOS (Not Line of Sight) and Multi-Path situations, the positioning accuracy and robustness of the UWB is hard to guarantee. In this article, only the line-of-sight propagation is taken into account.

The low-cost structure of the light scanner made for this project is composed of a structured light device emitting line laser (908 nm) and an infrared camera, fixed on a platform. The infrared camera is covered with an optical filter, which absorbs 99.99% of the visible light and transmits 80% of the infrared light at 908 nm, thus ensuring that the light of the laser is clearly displayed in the image. This design improves the quality of the image significantly and reduces the difficulty of image processing. More details about the structured light scanner are given in Section 4.

Ultrasound sensors are usually used for altitude control, but the errors of ultrasound sensors are hard to evaluate. Any obstacles on the floor, for example, would affect the accuracy of the sensors. In order to accurately evaluate the effectiveness of the proposed method, we did not employ ultrasound sensors.

## 4. Positioning Model

### 4.1. INS Model

**Figure 4:**
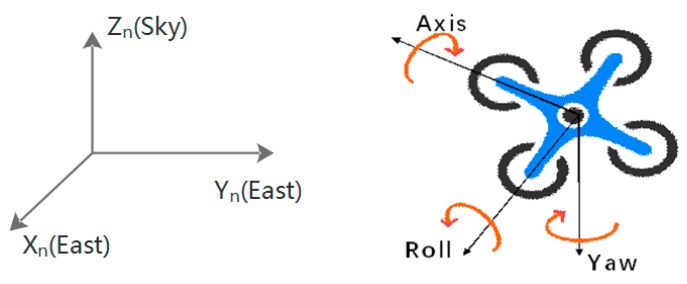
Navigation coordinate (**left**) and body coordinate (**right**) systems.

The first step to analyze the data from the IMU is to build a suitable coordinate system. Below are depicted two coordinate systems in which we are interested: the navigation coordinate system fixed to the earth and the body coordinate system fixed to the UAV, as illustrated in Figure 4.

The acceleration data collected from the IMU can be transformed from a body coordinate system to a navigation coordinate system with the following matrix:

(1) $$C_{b}^{n}=\left[\begin{array}{ccc} c\alpha \cdot c\beta & c\alpha \cdot s\beta \cdot s\gamma -s\alpha \cdot c\gamma & c\alpha \cdot s\beta \cdot c\gamma +s\alpha \cdot s\gamma \\ s\alpha \cdot c\beta & s\alpha \cdot s\beta \cdot s\gamma +c\alpha \cdot \gamma & s\alpha \cdot s\beta \cdot c\gamma -c\alpha \cdot s\gamma \\ -s\beta & c\beta \cdot s\gamma & c\beta \cdot c\gamma \end{array}\right]$$

where *c* = cos, *s* = sin, and γ, θ, and ψ represent the roll, pitch, and yaw, respectively. The gimbal lock problem is not taken into account here, as it is not likely to occur in our research.

Accelerations related to the navigation coordinate system are defined as:

(2) $$a_{n}=R_{b}^{n}a_{b}+G,$$

where

$$G=\left[\begin{array}{ccc} 0 & 0 & -g \end{array}\right]$$

and g is the gravity acceleration.

The position of the UAV may be predicted if the start position is known previously and accelerations are provided. As the IMU unit outputs discrete data, a position may be estimated by the following equation:

(3) $$v_{k}=v_{start}+\overset{k}{\underset{i=0}{\sum }}a_{n}^{\left(i\right)}∆T$$

(4) $$X_{k}=X_{start}+\sum_{i=0}^{k}\left[v_{i}∆T+\frac{1}{2}a_{n}^{\left(i\right)}∆T^{2}\right],$$

where v is the velocity and X is the position.

### 4.2. UWB Positioning Method

The UWB positioning system is composed of tag and anchor sensors. The system [24] estimates the 3D coordinates of the tag point using the TOA (Time of Arrival) technique by combining measurements from different anchor sensors. 

The position of the tag sensor is estimated using four fixed anchors at specified spots, and it is necessary to calculate the distance between tag and anchors with the following equation:

(5) $$d_{at}=c\left(t_{t}-t_{a}\right),$$

where c is the speed of light.

The relationship between the distance and the position of the sensors is written as:

(6) $$\{\begin{array}{c} {\left(x_{1}-x_{0}\right)}^{2}+{\left(y_{1}-y_{0}\right)}^{2}+{\left(z_{1}-z_{0}\right)}^{2}=d_{1}^{2}\\ \vdots \\ {\left(x_{4}-x_{0}\right)}^{2}+{\left(y_{4}-y_{0}\right)}^{2}+{\left(z_{4}-z_{0}\right)}^{2}=d_{1}^{2} \end{array}\;,$$

where $x_{i},y_{i},z_{i}(i=1,2,3,4)$ is the known position of the anchor sensors, and $x_{0},y_{0},z_{0}$ is the position of the tag sensors.

The above equation cannot produce an exact solution, but we can estimate the best solution with a minimum mean square estimate:

(7) $$\left({\hat{x}}_{0},{\hat{y}}_{0},{\hat{z}}_{0}\right)=\underset{\left(x_{0},y_{0},z_{0}\right)}{\min}\sum_{i=1}^{4}{\left[\sqrt{\left(x_{i}-x_{0}\right)+\left(y_{i}-y_{0}\right)+\left(z_{i}-z_{0}\right)}-d_{i}\right]}^{2}.$$

If the absolute location of the anchors is known in 3D space, then the 3D position of the tag sensor is also known. The estimated error mainly comes from errors in the measurement of the distance and the location of the anchors.

The precision of the distance measured depends on the precision of time. There are many ways to measure the time of the flight of a device. The synchronized method has a smaller time delay, but it has a higher demand for a chip, and it requires additional infrastructure. A non-synchronized transmitter and receiver method was used in the research reported in this paper. The method requires bidirectional communication between the tag sensor and anchor sensors. The tag sensor communicates individually with each of the anchor sensors to exchange timing information, in order to avoid the drawback that all sensors in the system must be time synchronized.

## 5. Principle of Structured Light Scanner

### 5.1. Overall of Structured Light Scanner

**Figure 5:**
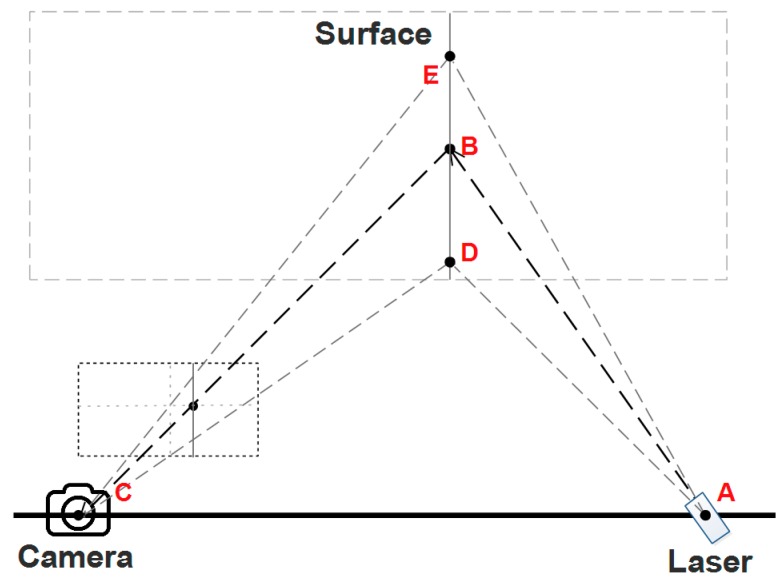
Principle of structured light scanner.

The principal of a structured light scanner based on triangulation is explained in Figure 5. The scanner projects a laser line onto the surface of the object, and a photo of the line is captured by a CMOS camera. As the laser is fixed onto the platform, the equation of the AED plane can be calculated through a calibration process. The equation of line CB, however, can be calculated by a perspective projection model. The position of point B then can be determined as the intersection of line CB with plane AED, and so on with the other points on the projected curve ED.

In order to retrieve the depth values for each point on the curve ED, which is a projection of the laser stripe on the object surface, we need to extract the projected position for each point on the curve ED in the image plane of the camera. Instead of an ideal curve, ED is actually a stripe with a certain width. Therefore, a skeletonization algorithm may be applied to extract the skeleton of the projected curve in the image plane. In this paper, we utilized a skeletonization algorithm based on FDT theory, and the details are described in the following section.

Once the depth value of the object is retrieved, we can utilize SLAM (Simultaneous localization and mapping) technology to determine localization using the device. The ICP (Iterative Closest Point) algorithm is adopted here to accomplish the localization, and it is hypothesized that the mapping of the environment is already known.

In order to get the positioning information, rotation matrix R and translation matrix T are calculated by matching the point cloud with the ICP algorithm, which minimizes the sum of the square error. The square error is defined as:

(8) $$E\left(R,T\right)=\frac{1}{N_{p}}\sum_{i=1}^{N_{p}}∥x_{i}-Rp_{i}-T^{2}∥,$$

where x is source point cloud, p is reference point and i is the number of point.

### 5.2. FDT Theory in Digital Cubic Space

The accuracy of the depth estimation depends mainly on the skeletonization algorithm. FDT theory is adopted here for the extraction of the skeleton. We used a dynamic programming–based algorithm to calculate the FDT values.

Let X be the reference set, then a fuzzy subset A of X is defined as a set of ordered pairs $A=\left\{\left(x,\mu_{A}\left(x\right)\right)|x\epsilon X\right\}$ where $\mu_{A}:X\to \left[0,1\right]$ is the membership function of A in X. A 2D fuzzy digital object O is a fuzzy subset defined on $Z^{2}$, i.e., $O=\left\{\left(p,\mu_{O}\left(p\right)\right)|p\epsilon Z^{2}\right\}$, where $\mu_{O}:Z^{2}\to \left[0,1\right]$. A pixel p belongs to the support of O if $\mu_{O}\left(p\right)>0$.

The fuzzy distance between two points p and q in a fuzzy object O is defined as being the length of the shortest path between p and q. The fuzzy distance $\Omega_{O}^{\left(1,\sqrt{2}\right)}:Z^{n}\times Z^{n}\to R$ between p and q in O is set to

$$\Omega_{O}^{\left(1,\sqrt{2}\right)}=min\langle p=p_{1},\cdots ,p_{m}=q\rangle =\sum_{i-1}^{m-1}\frac{1}{2}\left(\mu_{O}\left(p_{i}\right)+\mu_{O}\left(p_{i+1}\right)\right)\omega \left(p_{i+1}-p_{i}\right)$$

where $\omega \left(p_{i+1}-p_{i}\right)$ is the spatial Euclidean distance between $p_{i}$ and $p_{i+1}$, i.e., 1 if $p_{i}$ and $p_{i+1}$ are edge neighbors and $\sqrt{2}$ if they are vertex neighbors. We use $\Omega^{F}$ to denote the value of a point in a FDT and $\Omega_{O}$ for the distance function used to calculate a FDT. The Algorithm 1 is presented as follows.
**Algorithm 1:** Compute FDTInput: $O=\left(Z^{n},\mu_{O}\right)$
Output: an image ($Z^{n},\Omega$) representing FDT of O. 1. For all $p\epsilon \bar{\Theta \left(O\right)}$, set Ω(p) = 0; 2. For all pϵΘ(O), set Ω(p)=MAX; 3. For all pϵΘ(O) such that $N\left(P\right)\cap^{\text{​}}\bar{\Theta \left(O\right)}$ is not empty,   Push P into Q; 4. While Q is not empty do 5.    Remove a point P from Q; 6.    Find $dist_{min}=min_{q\epsilon N\left(p\right)}[\Omega \left(q\right)+w\left(p-q\right)\times \frac{1}{2}(\mu_{o}\left(p\right)+\left(\mu_{o}\left(q\right)\right]$
7.    If $dist_{min}<\Omega \left(p\right)$ then 8.        Set $\Omega \left(p\right)=dist_{min}$ 9.        Push all points $q\epsilon N\left(p\right)\cap^{\text{​}}\Theta \left(O\right)$ into Q 10. Output the FDT image O.

### 5.3. Experiments for FDT Theory

**Figure 6:**
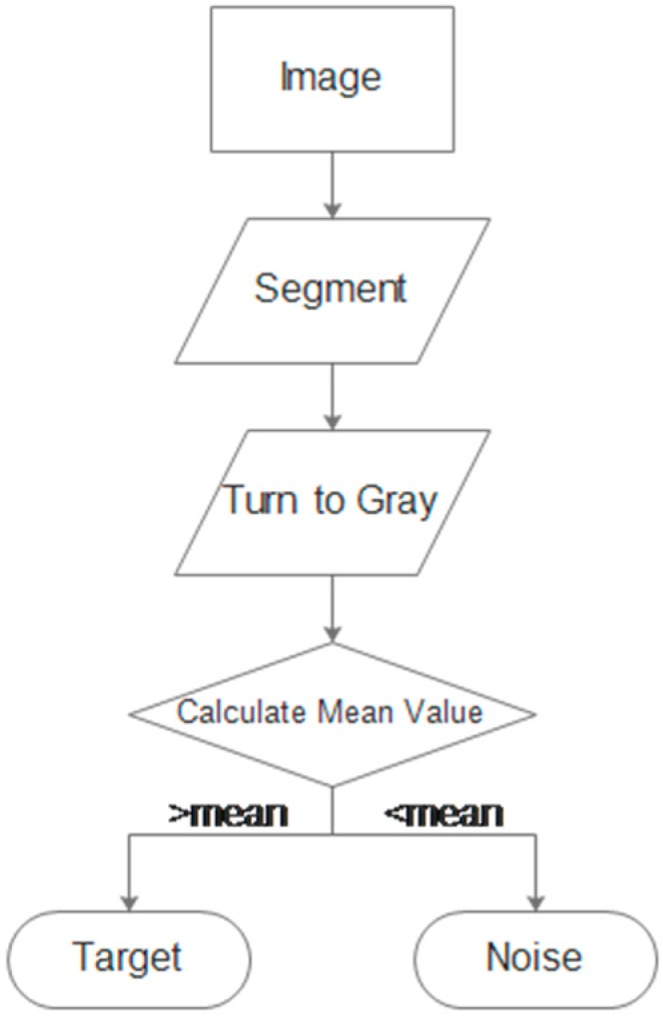
Image pre-processing steps.

Since an image contains only one light stripe, we first extract and segment the stripe from the image to reduce the computational complexity. Then, we enhance the contrast and convert the color image to gray. Finally, we distinguish between the target part and the background part with thresholds set by the mean intensity values of the image. The process is illustrated in Figure 6.

**Figure 7:**
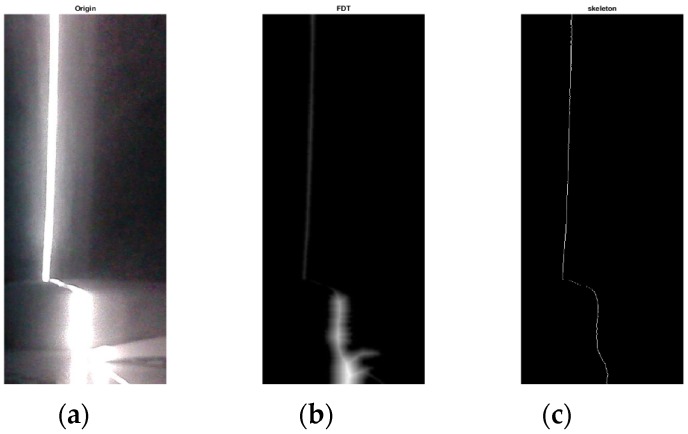
Result of skeletonization base on FDT. (**a**) The original image; (**b**) the FDT image; (**c**) the extracted skeleton of light stripe.

In this experiment, we verify the effectiveness of the proposed method based on FDT when extracting the skeleton of the light stripe. Compared with existing methods, the proposed algorithm can effectively deal with the highlighted part of the stripe due to the strong diffuse reflection. Figure 7 shows the skeletonization process, including the original image, the image after FDT, and the extracted skeleton of the light stripe. In order to evaluate the performance of different methods, we implemented three algorithms (FDT, DT, and Voronoi) to calculate the corresponding distance every five pixels along the skeleton and make a comparison reference to these distances.

**Figure 8:**
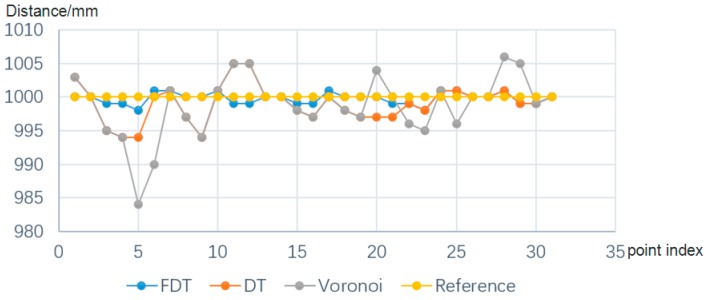
Distance measurement comparison.

Figure 8 illustrates the comparison result; we can see that the FDT curve is most similar to the reference curve, which implies that it is the best performance of the proposed FDT based method. The mean distance errors between the two corresponding pixels are shown in Table 1.

Furthermore, a structured light scanner will work only when the UAV hovers, as the tilt or motion of the UAV body would cause poor performance. Also, it only provides positioning information for two dimensions of the horizontal plane. Thus, it works as an auxiliary device to improve the positioning accuracy when the vehicle is in a hovering state.

## 6. Data Fusion Strategy

A data fusion algorithm, which fuses the data from the INS, UWB and structured light scanner, is proposed in this section to achieve better positioning accuracy.

**Figure 9:**
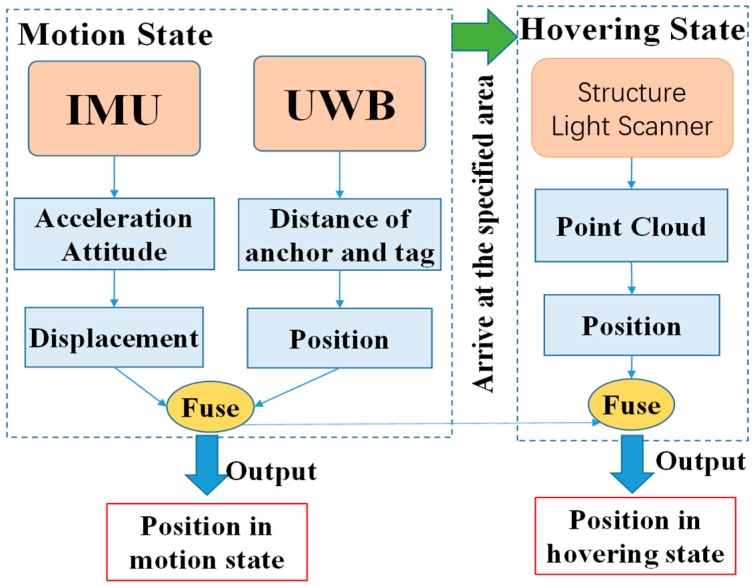
Data flow.

The overall information processing flow and data fusion strategy is illustrated in Figure 9. While the UAV is in motion, the system fuses the information of the IMU and UWB with an adaptive Kalman filtering system [25]. If the UAV arrives at the specified area, it will enter the hovering state to execute tasks, and the structured light scanner will be activated to ascertain the positioning information. The system then will fuse the data of the UWB and the structured light scanner with Gauss filtering in a hovering state.

### 6.1. Adaptive Kalman Filtering to Fuse the Data of INS and UWB in Motion State

A Kalman filter is a recursive estimator working in the time domain. It is hypothesized that the static function is linear and the noise accords with Gauss distribution. Only the prediction from the last time measurement, and the measurement of the current time, are required to produce the estimation for the current state. The algorithm works in a prediction phase and an update phase alternatively. The prediction phase predicts the state of the current time, and the update phase estimates the optimal state by incorporating the observation.

In a standard Kalman filter, the static matrix is:

(9) $$X_{t}=F_{t}X_{t-1}+B_{t}u_{t}+w_{t},$$

where $X_{t}$ denotes the estimate of the system's state at time step t, and $F_{t}$ is the state transition model. $B_{t}$ is the control-input model, which is applied to the control vector $u_{t}$. $w_{t}$ is the process noise, which is assumed to fit w~N(0,Q). The specific definitions are:

$$X=\left[\begin{array}{c} x\\ v \end{array}\right],$$

where x is the position, and v is the velocity;

$$F=\left[\begin{array}{cc} 1 & ∆T\\ 0 & 1 \end{array}\right]\;,$$

where ∆T is the time interval;

$$Bu=\left[\begin{array}{c} \frac{1}{2}a∆T^{2}\\ a∆T \end{array}\right],$$

where a is the acceleration.

The measurement matrix is:

(10) $$Z_{t}=H_{t}X_{t}+v_{t},$$

where $Z_{t}$ is the observation. $H_{t}$ is the observation model, which maps the true state space into the observed space. $v_{t}$ is the observation noise, which is assumed to be zero, indicating Gaussian white noise with covariance v~N(0,R). The specific definitions are:

$$H=\left[\begin{array}{cc} 1 & 0 \end{array}\right]$$

Adaptive Kalman filtering is developed based on conventional Kalman filtering to estimate the system noise variance-covariance matrix Q and measurement noise variance-covariance matrix R dynamically. This predicts the system and measurement noise from residual sequences. The residual sequence is defined as:

(11) $$c_{t}=Z_{t}-{\hat{Z}}_{t},$$

where $Z_{t}$ is the output of measurement, and ${\hat{Z}}_{t}$ is the prediction of measurement. ${\hat{Z}}_{t}$ is defined as:

(12) $${\hat{Z}}_{t}=H{\hat{X}}_{t},$$

where H is the measurement matrix, and ${\hat{X}}_{t}$ is the prediction of the state.

We assume that the measurement noise fits Gauss distribution, and then the probability density function of the measurement is:

(13) $$P_{{\left(z|\alpha \right)}_{t}}=\frac{1}{\sqrt{{\left(2\pi \right)}^{m}\left|C_{t}\right|}}e^{-\frac{1}{2}c_{t}^{T}C_{t}^{-1}c_{t}},$$

where α is the adaptive parameter, and m is the number of measurements. While the length of windows is N, the estimation based on the maximum likelihood is:

(14) $$\underset{\alpha }{\max}\sum_{j=j_{0}}^{t}P_{{\left(z|\alpha \right)}_{t}}=\underset{\alpha }{\min}\sum_{j=j_{0}}^{t}\ln\left(\left|C_{t}\right|\right)+c_{t}^{T}C_{t}^{-1}c_{t}.$$

After the differential operation, the expression is:

(15) $$\frac{\partial P}{\partial \alpha }=\sum_{j=j_{0}}^{t}\left[\mathrm{tr}\left\{C_{j}^{-1}\frac{\partial C_{j}}{\partial \alpha_{t}}\right\}-c_{t}^{T}C_{j}^{-1}\frac{\partial C_{j}}{\partial \alpha_{t}}C_{j}^{-1}c_{t}\right]=0.$$

Then, we can get the base function of the adaptive Kalman filtering based on the maximum likelihood:

(16) $$\sum_{j=j_{0}}^{t}\mathrm{tr}\left\{\left[C_{j}^{-1}-C_{j}^{-1}c_{t}c_{t}^{T}C_{j}^{-1}\right]\left[\frac{\partial R_{t}}{\partial \alpha_{t}}+H\frac{\partial Q_{t-1}}{\partial \alpha_{t}}H^{T}\right]\right\}=0.$$

In order to get the estimation value of $R_{t}$, we assume that the measurement noise $Q_{t}$ is independent of the adaptive parameter α and the value of $Q_{t}$ is known. Let $\alpha_{i}=R_{ii}$, and then we can get:

(17) $$\sum_{j=j_{0}}^{t}\mathrm{tr}\left\{C_{j}^{-1}\left[C_{j}-c_{t}c_{t}^{T}\right]C_{j}^{-1}\right\}=0.$$

Then, the estimation value of $R_{t}$ is:

(18) $${\hat{R}}_{t}={\hat{C}}_{t}-H{\hat{P}}_{t}H^{T}.$$

As with the measurement noise, we assume that the system noise $R_{t}$ is independent of the adaptive parameter α, and the value is known. Let $\alpha_{i}=Q_{ii}$, and then we can get:

(19) $$\sum_{j=j_{0}}^{t}\mathrm{tr}\left\{H^{T}\left[C_{j}^{-1}-C_{j}^{-1}c_{t}c_{t}^{T}C_{j}^{-1}\right]H\right\}=0.$$

The estimation value of $Q_{t}$ is:

(20) $${\hat{Q}}_{t}=K{\hat{C}}_{t}K^{T}.$$

After getting the estimation value of $Q_{t}$ and $R_{t}$, we can apply these values to the recursive process of the Kalman filtering.

### 6.2. Gauss Filtering to Fuse the Data of the UWB and the Structured Light Scanner in the Hovering State

According to the convolution property that two Gaussians convolve to make another Gaussian [26], the result of new Gaussian form integrating the UWB and the structured light scanner can be written as:

(21) $$f_{fused}\left(x\right)=\frac{1}{\sqrt{2\pi \sigma_{fused}^{2}}}e^{-\frac{{\left(x-\mu_{fused}\right)}^{2}}{2\sigma_{fused}^{2}}},$$

where

(22) $$\mu_{fused}=\mu_{u}+\frac{\sigma_{u}^{2}\left(\mu_{l}-\mu_{u}\right)}{\sigma_{u}^{2}+\sigma_{l}^{2}}$$

and

(23) $$\sigma_{fused}=\sqrt{\sigma_{u}^{2}-\frac{\sigma_{u}^{4}}{\sigma_{u}^{2}+\sigma_{l}^{2}}}.$$

We can utilize the above equation to fuse the data of the UWB and the structured light scanner. Compared to Kalman filtering, this is easier to understand and could easily fuse multiple sources of data with a simple mathematical model.

## 7. Experimental Works of Data Fusion

### 7.1. Experiment in Motion State

**Figure 10:**
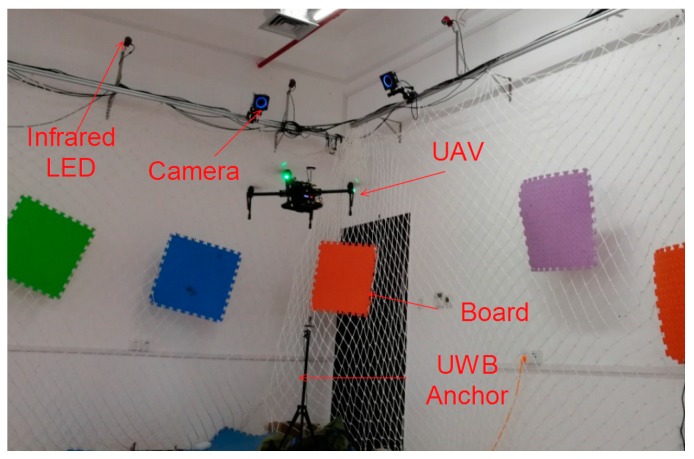
Experiments with a motion capture system [27].

In this section, an experiment on a device in motion is described to verify the performance of the proposed data fusion strategy. In order to determine the real path of the UAV, the experiment is carried out in a lab equipped with a motion capture system, as shown in Figure 10.

**Figure 11:**
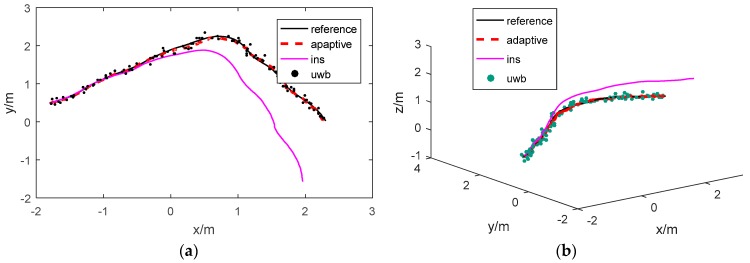
(**a**) Path tracking (x and y); (**b**) path tracking (x, y, and z).

The experiment results are presented in Figure 11a, which presents the path along the x and y axes for the convenience of comparison, and also in Figure 11b, which displays the results in three-dimensional space. As shown in the figures, the real path is tracked by a motion capture system, and it is plotted in a black line as a reference. The other three paths are calculated using the INS, UWB and adaptive Kalman, respectively. The updating frequency of the IMU is 100 Hz, and the UWB is 10 Hz. The adaptive Kalman filtering fuses the information only from the INS and UWB. 

As shown in Figure 11a, the path estimated by the proposed strategy is more accurate than that estimated by the INS and UWB independently. INS updates position data at a high refresh rate, and its error is relatively small over a short period of time, but the results diverge quickly. The inherent defects that occur as the errors accumulate make it impossible for the device to work alone for a long period of time. Thus, the UWB positioning technique with a margin of error that is less than 15cm may be used to correct the accumulated error of the INS.

**Figure 12:**
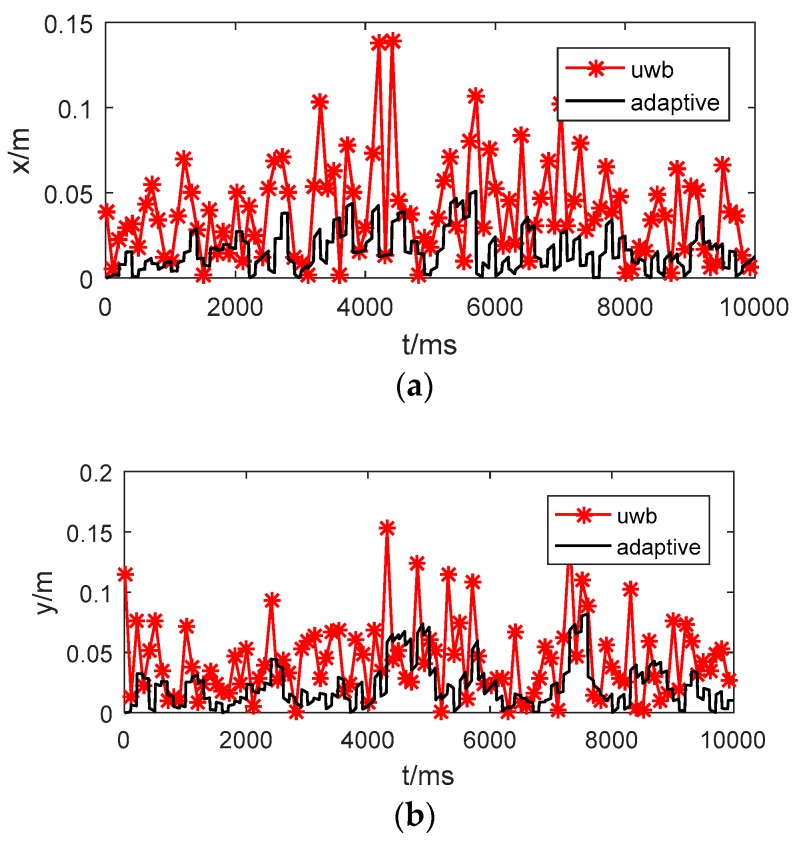

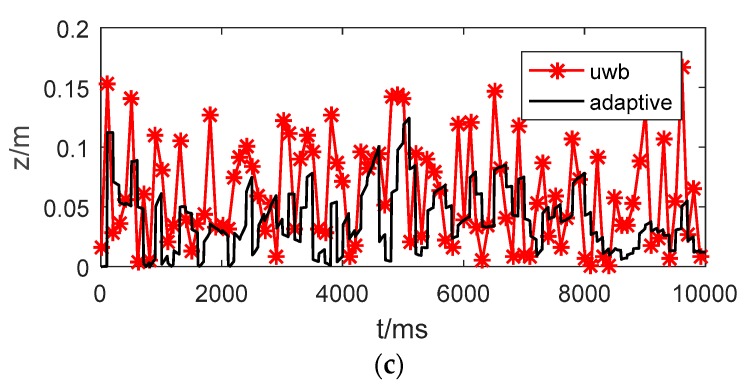
Position errors of the UWB and proposed strategy in three directions. (**a**) Position error along the x axis; (**b**) position error along the y axis; (**c**) position error along the z axis.

Figure 12 presents the absolute position error of the UWB and adaptive Kalman filtering along the x, y and z axes. The mean errors of the different methods are listed in Table 2. We may observe that the performance of adaptive Kalman filtering is best. To evaluate robustness, the maximal errors are presented in Table 3. The method based on adaptive Kalman filtering provides the highest positioning accuracy, with errors as low as 10 cm along the x and y axes and 15 cm along the z axis in the experiment. Applying a low-pass filtering method may further improve the experimental results in particular cases. However, it could also filter out certain useful high frequency signals that would affect the robustness of the system. 

### 7.2. Experiment in the Hovering State

**Figure 13:**
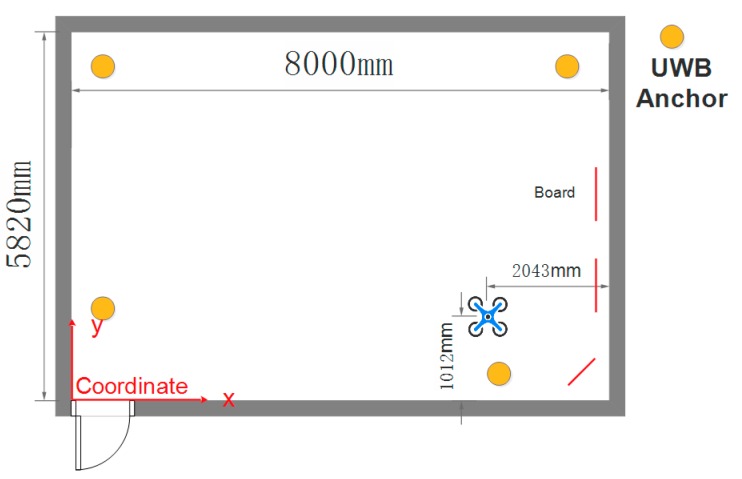
Experimental setup in hovering state.

While the UAV is hovering in a specified area, the structured light scanner may be useful to obtain a more accurate position. Gauss filtering is applied to fuse the data from the UWB and the structured light scanner. The experiment environment is illustrated in Figure 13. The real coordinates of the UAV are (5957.8 mm, 1012.1 mm).

**Figure 14:**
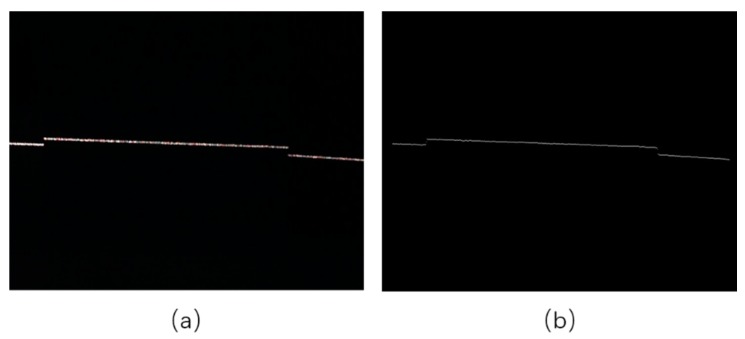
(**a**) Image captured by camera; (**b**) skeleton.

Figure 14a presents the image captured by the structured light scanner, and Figure 14b is the extracted skeleton.

The equation for the scanning plane of the laser stripe is:

9.98509x+0.431365y+0.334608z+823.797=0.

**Figure 15:**
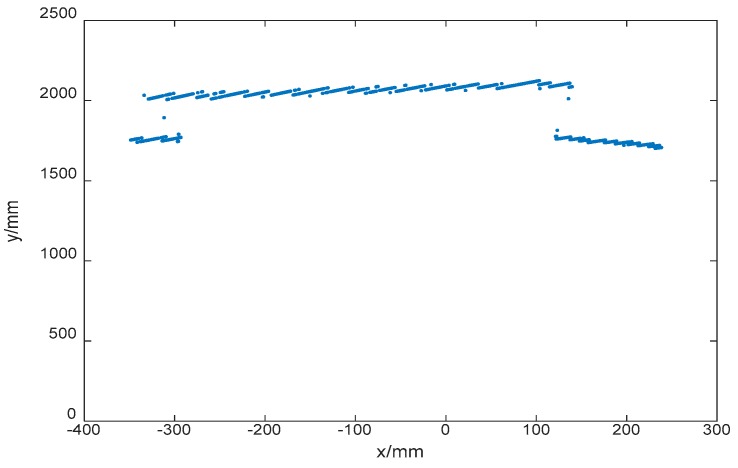
Depth map.

The depth map is calculated and shown in Figure 15. After that, an iterative algorithm ICP is adopted to match the point cloud, and the coordinates of UAV are determined as (5937.41 mm, 997.97 mm), and the error is (−20.39 mm, −14.13 mm).

We conducted 10 experiments in different places. The mean and maximal positioning errors for the different methods are listed in Table 4 and Table 5.

The structured light scanner can provide more accurate position estimations than the UWB; meanwhile the fused method is better than the structured light scanner-based method. As the noise of the structured light scanner does not strictly accord with Gauss distribution, the performance is improved moderately. The maximal error of the proposed method, however, is smaller. Compared to the performance in motion, it is a significant improvement.

### 7.3. Comparison with Other Methods

The method of indoor positioning varies, as the requirements are different. Li et al. [28] implements the LIDAR/MEMS IMU Integrated Navigation (SLAM) Method for a small UAV in indoor environments with an error margin of approximately 0.5 m. In [13], with the UWB, a 95% probability positioning accuracy of 10 cm in the horizontal plane and 20 cm in the three-dimensional space was experimentally achieved. The indoor positioning accuracy achieved by the method proposed in this paper can reach a level within 15 cm while the device is in motion, as well as 5 cm when the device is in the hovering state in the horizontal plane, and within 20 cm along the z axis.

## 8. Conclusions

Choosing sensors for the localization of UAVs for indoor navigation can be a difficult task. In this paper, a setup including an INS, UWB, and structured light scanner is proposed. An optimized data fusion strategy is designed to improve the positioning accuracy in the moving and hovering states. The simulation results show that the INS cannot work for a long period of time without cooperation with the UWB. The integration of the INS and UWB can improve the positioning accuracy effectively. Moreover, we made a structured light scanner to retrieve a depth map and utilized an FDT algorithm to improve its performance. The structured light scanner can retrieve high-precision data about the position of the UAV around the target point and help to accomplish specified tasks effectively. Therefore, it can be used as an auxiliary device for high-precision positioning when the UAV is in a hovering state. With the proposed method, we achieved an accuracy of 15 cm for a device in motion and 5 cm while it is in a hovering state.

Currently, we have only tested our method in a simple environment. A complex environment remains a challenge for accurate positioning. The influence of the space configuration could be important in more complex cases, and the positioning accuracy of the UWB would be unstable. In future work, we will conduct more research on the UWB and try to upgrade our setup and methods to make them adaptable to complex environments. The structured light scanner is also expected to work for devices in motion, while the data fusion strategy will be further optimized for real-world applications. Also, the orientation is important in many applications. In this paper, we focus mainly on the positions of the UAV; therefore, evaluating the errors of orientation will be carried out in future work. 

## Figures and Tables

**Table 1:** Mean distance errors with three different methods.

Method	FDT	DT	Voronoi
Errors (mm)	1.01	1.76	5.10

**Table 2:** Mean errors of different methods.

Mean Error	x (m)	y (m)	z (m)
INS only	0.366	0.150	1.03
UWB only	0.056	0.056	0.074
Adaptive Kalman	0.027	0.02	0.036

**Table 3:** Maximum errors of different methods.

Maximal Error	x (m)	y (m)	z (m)
UWB only	0.141	0.157	0.175
Adaptive Kalman	0.052	0.083	0.124

**Table 4:** Mean errors for different methods.

Mean Error	x (m)	y (m)
UWB	0.0484	0.0407
Structured light scanner	0.0238	0.0211
Fused method	0.0199	0.0175

**Table 5:** Maximal errors for different methods.

Maximal Error	x (m)	y (m)
UWB	0.0985	0.0853
Structured light scanner	0.0496	0.0384
Fused method	0.0379	0.0250
